# The Role of Sigmar1 in Autophagy Regulation and Disease Therapy

**DOI:** 10.3390/ijms27104492

**Published:** 2026-05-17

**Authors:** Huanqing Ge, Yusi Lin, Junda Li, Renwen Zhang, Cangcang Xu

**Affiliations:** Key Laboratory of Study and Discovery of Small Targeted Molecules of Hunan Province, School of Pharmaceutical Sciences, Health Science Center, Hunan Normal University, Changsha 410013, China; 15207367161@163.com (H.G.); lin.yusi@outlook.com (Y.L.); 202230191103@hunnu.edu.cn (J.L.); zrrr666@163.com (R.Z.)

**Keywords:** Sigmar1, autophagy, regulatory mechanism, agonists and antagonists, disease therapy

## Abstract

Sigmar1 is a multifunctional molecular chaperone protein located on the Mitochondria-associated endoplasmic reticulum membranes (MAM). Recent studies have shown that Sigmar1 is not only a regulatory protein involved in cellular stress responses but also plays a significant role in the process of autophagy. It regulates the initiation and progression of autophagy by influencing multiple autophagy-related signaling pathways and interacting with key proteins such as LC3 and GABARAP. This regulation exhibits a dual nature. On one hand, it can induce protective autophagy, helping cells cope with stress such as oxidative stress and endoplasmic reticulum stress, thereby playing a protective role in the progression of diseases such as neurodegenerative disorders and cardiovascular diseases. On the other hand, in certain cancers, Sigmar1 may also promote tumor cell survival through autophagy regulation, thereby exacerbating disease progression. Consequently, developing agonists and antagonists targeting Sigmar1 has become a highly promising therapeutic strategy. This review provides a systematic overview of recent advances in the biological characterization of Sigmar1 and its molecular mechanisms in regulating autophagy. It summarizes the multifaceted roles of Sigmar1 in various diseases and discusses current research progress and the application prospects of Sigmar1 agonists and antagonists, aiming to establish a theoretical foundation for the development of novel Sigmar1-based therapeutic strategies for human diseases.

## 1. Introduction

Autophagy is a crucial process for maintaining cellular homeostasis, playing a significant role in enabling cells to cope with oxidative stress, nutrient deprivation, and adapt to environmental changes by degrading abnormal proteins and damaged organelles. Its deregulation has been confirmed as a pathological basis for various major diseases, including neurodegenerative diseases, cancer, and cardiovascular disease [1,2]. Therefore, investigating the precise regulatory network of autophagy has become a forefront focus in current life science and medical research.

The discovery of Sigmar1 provides a novel perspective for studying autophagy regulation. As a molecular chaperone protein located at the MAM, Sigmar1 is extensively involved in physiological processes such as cellular stress responses, ion channel regulation, and mitochondrial function maintenance [3,4]. A growing body of research suggests that Sigmar1 may serve as a key molecule in autophagy, exerting regulatory functions across multiple stages including autophagy initiation, autophagosome formation and maturation, and autolysosomal degradation. However, several issues in this field remain to be clarified. First, the molecular mechanisms by which Sigmar1 regulates autophagy are complex and not yet fully elucidated, entailing the need for systematic integration of cross-talking signaling pathways. Second, the role of Sigmar1-mediated autophagy differs across various disease models. For instance, in neurodegenerative diseases such as AD, it may delay disease progression by inducing autophagy, whereas in certain cancers, it might promote malignant progression by enhancing autophagy [5,6]. The decisive factors underlying this dual effect remain unclear. Finally, the therapeutic prospects and limitations of Sigmar1-targeted agonists and antagonists, as potential tools for modulating autophagy and treating related diseases, require comprehensive evaluation.

Based on the above background, this review aims to systematically summarize the mechanisms by which Sigmar1 regulates autophagy and the latest research progress in related diseases. Through a deep analysis of the molecular mechanisms of the Sigmar1-autophagy axis and a comprehensive elucidation of its functions under various pathological conditions, as well as an exploration of the application potential of agonists and antagonists targeting Sigmar1, we expect to provide direction for future basic research and drug development targeting Sigmar1.

## 2. The Structure and Function of Sigmar1 Protein

### 2.1. Structure of Sigmar1 Protein

**Figure 1:**
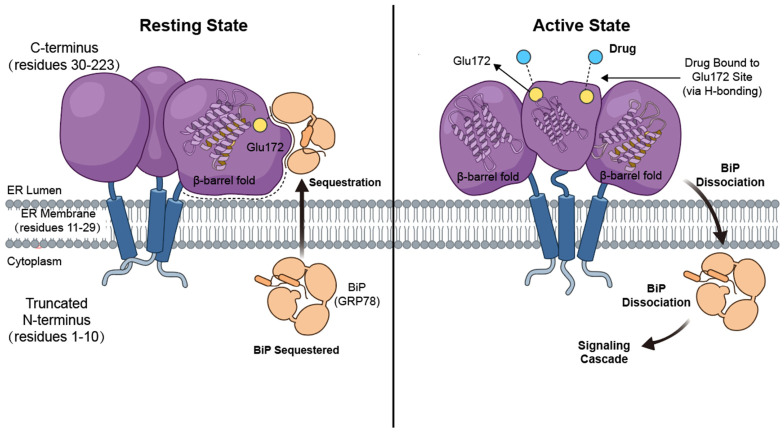
The Structure of Sigmar1 protein.

Sigmar1 is a highly conserved, ubiquitously expressed molecular chaperone that is encoded by *SIGMAR1* gene and predominantly localized to the MAM [7]. It exists in multiple oligomeric states, with the homotrimer representing the minimal stable oligomeric unit. Topologically, the full-length 223-amino acid structure of this single-pass transmembrane protein comprises three core domains [8]. The C-terminal (residues 30–223) domain contains a cupin-like β-barrel, the cavity of which constitutes the primary ligand-binding site, and features a flat hydrophobic surface that facilitates the enrichment and anchoring of Sigmar1 at the MAM [9]. It is entirely compartmentalized within the ER lumen and dictates the vast majority of the protein’s pharmacological functions [10]. Within the ligand-binding pocket, the highly conserved Glu172 residue serves as the core anchoring site for most small-molecule drugs via hydrogen bonding [11]. Furthermore, its extensive and flat hydrophobic surface is closely associated with the luminal face of the ER membrane, providing a critical interaction and dissociation region for the ER chaperone BiP (GRP78). Under resting cellular conditions, BiP is tightly sequestered with the Sigmar1 C-terminus. Upon the specific binding of a pharmacological ligand to this pocket, the cellular conditions are activated and the dissociation of BiP from the receptor is induced. Consequently, essential downstream intracellular signaling cascades are activated by the liberated BiP [8,12,13]. The N-terminal (residues 1–10) domain contains an ER retention motif that ensures the proper insertion of Sigmar1 into the ER membrane, thereby facilitating its enrichment at the MAM [4]. Additionally, the truncated, cytoplasm-facing N-terminus is accompanied by a single hydrophobic transmembrane helix that traverses the ER membrane (residues 11–29) (Figure 1).

### 2.2. Localization and Expression of Sigmar1

Sigmar1 is mainly enriched at the MAM, which are the sites of tight contact sites between the ER membrane and the mitochondrial outer membrane that are connected by protein tethers [7,14]. Sigmar1 is ubiquitously expressed throughout various tissues, with predominant expression in the liver, brain, and heart [15,16]. Sigmar1 is widely expressed across diverse cell types, including neurons, hepatocytes, and cardiomyocytes, which is consistent with its pleiotropic roles in various physiological and pathological processes, ranging from cellular stress responses and autophagy modulation to metabolic regulation. For instance, in neurons, Sigmar1 exhibits neuroprotective properties and anti-amnesic effects [17]; in hepatocytes, it is involved in lipid metabolism and steatosis [18]; and in cardiomyocytes, Sigmar1 expression maintains mitochondrial respiratory homeostasis and confers cardioprotective effects [19,20]. Recent investigations into the subcellular localization of Sigmar1 have revealed that its agonists promote the lateral mobility of Sigmar1 confined to the ER membrane, challenging the earlier model of extensive inter-organelle trafficking in response to activation [21], suggesting Sigmar1 dynamics may depend on cell types.

### 2.3. Functions of Sigmar1 Protein

#### 2.3.1. Physiological Functions

Sigmar1 plays critical roles in diverse cellular physiological processes, including the modulation of calcium channel signaling [13,22], the regulation of mitochondrial morphodynamics and respiratory function [19], the mediation of cellular stress response [13], and the regulation of autophagy and lipid metabolism [23]. Furthermore, recent studies have revealed that Sigmar1 oligomers utilize their extended amphipathic helical arrays to engage the ER membrane from the luminal side, thereby physically flattening the membrane to stabilize planar ER sheet structures [24].

#### 2.3.2. Dysfunction

Aberrant expression or dysfunction of Sigmar1 contributes to the pathogenesis of various diseases through the dysregulation of cellular stress responses, the disruption of signal transduction, and the interference with organelle interactions. A growing body of evidence has implicated Sigmar1 in the pathogenesis of numerous diseases. In the context of neuromuscular diseases, the c.238C>T (p.Arg80Ter) and c.151+1G>T (p.31_50del) mutations in the *Sigmar1* gene have been shown to promote motor neuron-like cell degeneration, thereby contributing to the development of distal hereditary motor neuropathy [25]. In neurodegenerative disorders, Sigmar1 inactivation exacerbates the progression of AD [26]. In psychiatric disorders, activation of Sigmar1 alleviates methamphetamine-induced anxiety and depression [27]. In cardiovascular diseases, Sigmar1 antagonism exacerbates heart failure following myocardial infarction, whereas Sigmar1 agonism significantly attenuates post-infarction heart failure through pro-angiogenic effects [28]. In malignancies, Sigmar1 antagonism triggers lipophagy, thereby suppressing prostate cancer cell proliferation [29]. In primary breast cancer, Sigmar1 is highly expressed and correlates with invasive status [30]. In summary, Sigmar1 emerges as a promising therapeutic target for modulating the progression of multiple diseases.

## 3. Processes and Critical Regulatory Molecules in Cellular Autophagy

Autophagy, also termed macroautophagy, is a highly conserved process through which intracellular material is delivered to the lysosome for degradation [31,32]. Within the cell, a double-membrane structure termed the autophagosome is formed. This structure engulfs cytoplasmic cargo and subsequently fuses with the lysosome, where acid hydrolases degrade the sequestered material, thereby facilitating cellular self-digestion [33]. In addition to macroautophagy, microautophagy and chaperone-mediated autophagy also occur in cells [34].

### 3.1. Autophagy Initiation and Progression

Autophagy occurs through four distinct phases: (1) initiation of the autophagic pathway; (2) formation of the phagophore and subsequent generation of the autophagosome; (3) fusion of the autophagosome with the lysosome to form an autolysosome; and (4) degradation of the engulfed substrates within the autolysosome. Autophagy is initiated in response to various autophagy-initiating signals, such as those mediated by the mTOR and AMPK pathways. Upon initiation, a double-membrane structure known as the phagophore emerges in the cytoplasm. Through the coordinated actions of Atg protein complexes (including the ULK1 kinase complex and the Vps34–Beclin1 complex) and LC3-related proteins (mammalian Atg8 homologs), the phagophore gradually elongates, engulfing cytoplasmic cargo before ultimately sealing to form a mature autophagosome. Following intracellular transport, the autophagosome fuses with a lysosome to generate an autolysosome, where the sequestered cargo is degraded by hydrolytic enzymes [33,35].

Microautophagy is a process whereby cytoplasmic material is directly sequestered and degraded by the lysosomal membrane through invagination, protrusion, or related mechanisms, and occurs independently of autophagosome formation. Chaperone-mediated autophagy is a selective lysosomal degradation pathway in which the chaperone protein HSPA8 recognizes cytosolic proteins containing a KFERQ-like motif, targets them to LAMP2A, and facilitates their translocation into the lysosome for degradation, a process that occurs without the formation of vesicles [34].

### 3.2. Critical Regulators of Autophagy

Autophagy is regulated at multiple levels, with mTOR, LC3, and the Vps34–Beclin1 complex serving as critical regulators of this process.

The mTOR signaling pathway is considered central to the regulation of autophagy. In mammals, mTOR is found in two distinct complexes, mTORC1 and mTORC2, with mTORC1 suppressing autophagy by phosphorylating and inactivating ULK1 [36]. Autophagy can be induced by rapamycin, which suppresses mTORC1 activity by binding to its Raptor subunit [37]. mTORC1 is also regulated by upstream signaling pathways including AMPK and Akt [38].

LC3 plays a critical role in autophagosome formation. The LC3 precursor protein undergoes proteolytic processing and lipidation, resulting in the sequential formation of LC3-I and LC3-II. LC3-II is anchored to the elongating autophagosomal membrane and is involved in promoting autophagosome biogenesis. In addition, LC3-II can bind indirectly to cargo destined for degradation through selective autophagy receptor proteins such as p62/SQSTM1, a process that mediates selective autophagy [39].

The class III PI3K (Vps34)–Beclin1 complex is also considered essential for autophagosome membrane formation. This complex is responsible for the generation of PI3P, which recruits downstream autophagy proteins such as Atg18, thereby promoting elongation of the phagophore membrane [40]. Beclin1, as a core regulatory subunit, is subject to inhibition by Bcl-2 family proteins. Extrinsic stimuli such as nutrient deprivation can alleviate this inhibition, thereby activating autophagy [41].

In recent years, the molecular mechanisms of cellular autophagy have been elucidated through major breakthroughs. It has become evident that the initiation and progression of autophagy are not limited to classical pathways but are instead subject to fine regulation through multiple novel mechanisms. For instance, during autophagosome recognition and formation, GABARAP proteins have been shown to recognize and stabilize the synaptic development protein ARMS through non-canonical, LIR-motif–independent mechanisms, a finding that expands the understanding of autophagy in neuron-specific functions [42]. At the critical step of autophagosome membrane sealing, the Atg4 protease has been shown to catalyze the delipidation of Atg8 family proteins from the membrane, acting in parallel with the ESCRT membrane remodeling complex to ensure autophagosome integrity [43]. At the level of lysosomal fusion and regeneration, the newly identified Gal3–CaN–Smurf1 complex has been shown to sense lysosomal damage and activate the master transcriptional regulator TFEB by alleviating mTORC1-mediated inhibition and promoting ubiquitination, a process that ultimately triggers lysosomal regeneration and the transcription of autophagy-related genes [44].

## 4. The Regulation of Autophagy by Sigmar1

**Figure 2:**
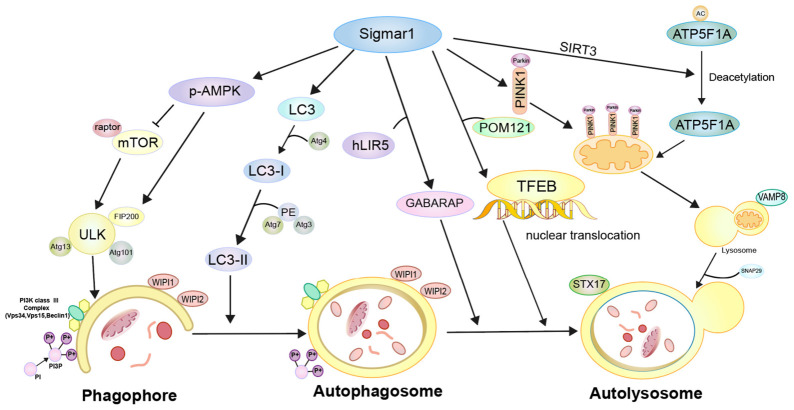
The role of sigmar1 in autophagy regulation.

Recent studies have shown that Sigmar1 plays an important role in all stages of autophagy, including the initiation stage, the formation and elongation of the autophagosome, as well as the formation and degradation of the autolysosome (Figure 2).

### 4.1. Association Between Sigmar1 and Signaling Pathways Involved in Autophagy Initiation

AMPK is a serine/threonine kinase that negatively regulates lipid anabolism. As a key energy-sensing kinase within cells, AMPK activates various catabolic processes, such as glucose uptake and metabolism, while simultaneously inhibiting several anabolic pathways, including the synthesis of lipids, proteins, and carbohydrates [45]. Under basal conditions, AMPK remains inactive. It can be directly phosphorylated and activated by upstream kinases such as LKB1, CaMKKβ, and TAK1 [46,47,48]. Alternatively, under stress conditions such as hypoxia or glucose deprivation, cellular energy levels decrease, leading to an increased AMP/ADP ratio, which subsequently activates AMPK. Following its activation, AMPK has been shown to directly phosphorylate ULK1 at residues such as Ser555 and Ser777 [49], thereby initiating autophagy. AMPK can also induce autophagy by inhibiting mTORC1 activity through the LKB1-AMPK signaling axis [50]. Following its induction, autophagy proceeds with the formation of the ULK1 complex, which consists of ULK-Atg13-FIP200-Atg101 [51]. This complex serves as a bridge that connects upstream signals to downstream autophagosome formation [52,53] and plays a critical role in the initial stages of autophagy. When cellular energy levels are sufficient, mTORC1 binds to the ULK1 complex, phosphorylates Atg13 and ULK1, and thereby inhibits autophagy initiation. Under starvation conditions, mTORC1 dissociates from the complex, leading to the dephosphorylation of Atg13 and FIP200, a process that promotes autophagy initiation [54,55,56]. It has been shown that pathogenic mutations in *Sigmar1* (C238T and 31_50del) upregulate the phosphorylation levels of AMPK and ULK1, enhance the expression of autophagic flux markers such as LC3B and Atg7, and reduce p62 levels in dHMN. Following AMPK knockout, *SIGMAR1* mutation-induced cellular autophagy and apoptosis were significantly reduced, indicating that mutant Sigmar1 promotes autophagy by activating the AMPK/ULK1 pathway [25]. Another study on sepsis-associated acute kidney injury revealed that a decrease in Sigmar1 leads to reduced phosphorylation of AMPK and ULK1, alongside increased levels of phosphorylated mTOR. Conversely, upregulation of Sigmar1 enhances the phosphorylation levels of AMPK and ULK1 while suppressing the phosphorylation of mTOR, suggesting that Sigmar1 promotes autophagy through the AMPK/mTOR signaling pathway [57].

### 4.2. Sigmar1’s Impact on Autophagosome Formation and Elongation

The formation of autophagosomes is a critical step in the autophagy process. During the initial stages of autophagosome formation, the class III PI3K complex constitutes another essential multi-protein signaling complex that acts downstream of the ULK1 complex (ULK-Atg13-FIP200-Atg101). This complex, which is composed of Vps34, Vps15, and Beclin1 (Atg6), is localized to the phagophore assembly site, where it binds to membrane lipids and is responsible for PI to generate PI3P [58,59]. PI3P subsequently recruits downstream effector proteins including Atg18 and Atg21 to the phagophore membrane [60]. WIPI1 and WIPI2 are recognized as mammalian homologs of Atg18. During the initial phase of autophagosome formation, WIPI2 is recruited by Atg16L1 and ULK1 to the nascent autophagosome, where it participates in the lipidation of LC3. Meanwhile, WIPI1 binds to PI3P via a specific FRRG motif located within its β-sheet structure, thereby contributing to autophagosome formation and subsequent fusion with lysosomes [61,62,63]. The elongation of the autophagosome is primarily dependent upon two ubiquitination systems: the Atg12-Atg5 system and the Atg8/LC3 system. These two systems function in a coordinated manner to regulate one another and play critical roles during autophagy [64]. The mammalian Atg8 family of proteins include LC3A, LC3B, and GABARAP, among others [39]. LC3 first undergoes dehydroxylation by Atg4, resulting in the formation of cytoplasmic LC3-I. Subsequently, mediated by Atg7 and Atg3, LC3-I is conjugated with the lipid PE, thereby forming the lipidated LC3-II, which is anchored to the autophagosomal membrane. This conversion has established LC3 as a key marker for monitoring autophagy [2]. It has been demonstrated by Knupp et al. [65] that Sigmar1 binds to the 3′ untranslated region (3′ UTR) of LC3B mRNA, recruits it to ER-associated structures, and promotes the local translation and functional lipidation of LC3, a mechanism that regulates the initiation of functional autophagy. These findings indicate that Sigmar1 regulates the progression of autophagy by promoting LC3 lipidation, thereby influencing autophagosome formation and elongation.

### 4.3. The Impact of Sigmar1 on Autolysosome Formation and Degradation

Following autophagosome formation, fusion with a lysosome occurs to generate an autolysosome. The fusion is typically regulated by SNARE, which includes the t-SNARE protein STX17, the synaptophysin VAMP8, and the synaptosome-associated protein SNAP29 and others [66]. STX17, localized on mature autophagosomal membranes, interacts with lysosomal membrane-associated VAMP8 via cytosolic SNAP29 to facilitate membrane fusion and autolysosome formation [67]. During the degradation phase, both the inner membrane and the luminal contents of the autophagic vesicle are degraded by lysosomal enzymes. The TFEB, a key regulator of lysosomal function in this process, is phosphorylated by mTORC1. This phosphorylation promotes TFEB nuclear translocation, where it induces transcription and translation of lysosomal and autophagy-related genes [68,69]. It was revealed by Wang et al. [70] that Sigmar1, acting as a molecular chaperone, directly binds to the nucleoporin POM121. Consequently, POM121 recruits KPNB1/importin β1, facilitating TFEB nuclear translocation and thereby activating the expression of autophagy-related genes. Another research by Lin et al. [71] demonstrated that fluvoxamine activates Sigmar1, stabilizes the expression of nucleoporin POM121, promotes the nuclear translocation of TFEB, and thereby elucidates the molecular mechanism underlying the amelioration of autophagic dysfunction in ALS models. GABARAP, a member of the Atg8 protein family, is also involved in autophagy-related processes. GABARAP-II colocalizes with LC3-II and functions in autophagy [72], with GABARAP considered more specifically involved in autophagosome–lysosome fusion [73,74]. A recent study identified a specific interaction between Sigmar1 and GABARAP via its fifth LC3-interacting region (hLIR5: 81WVFV84). Activated Sigmar1 is anchored to the autophagosomal membrane through hLIR5, promoting local enrichment of GABARAP. As GABARAP is a key regulator of autophagosome–lysosome fusion, Sigmar1 may directly participate in the assembly of the STX17/VAMP8 fusion machinery by stabilizing GABARAP membrane localization, although the precise mechanism remains unclear [75]. Collectively, these findings indicate that Sigmar1 plays a role in autophagosome formation and degradation within the autolysosomal pathway, a process that is critical for the regulation of autophagy.

### 4.4. The Impact of Sigmar1 on Selective Autophagy

Mitophagy is a selective autophagic process by which damaged mitochondria are eliminated, and is primarily mediated by two distinct mechanisms: the ubiquitin-dependent pathway, exemplified by the PINK1/Parkin axis, and ubiquitin-independent pathways, which rely on mitochondrial outer membrane receptors such as NIX, BNIP3, and FUNDC1. In the PINK1/Parkin pathway, loss of mitochondrial membrane potential leads to the accumulation of PINK1 and subsequent activation of Parkin. Activated Parkin ubiquitinates damaged mitochondria, thereby recruiting autophagy adaptors such as OPTN and NDP52 and ultimately targeting these organelles for degradation by the autophagosome–lysosome system [76,77,78]. In the ubiquitin-independent pathway, autophagy is initiated through the direct binding of LC3 to outer mitochondrial membrane proteins [79,80].The regulatory role of Sigmar1 in PINK1/Parkin-mediated mitophagy was first reported by Wang et al. [81], who demonstrated that the Sigmar1 agonist PRE-084 significantly attenuated dopaminergic neuron loss and motor dysfunction while restoring impaired mitophagic activity. Conversely, Sigmar1 knockdown or knockout was found to exacerbate both mitophagic defects and cytotoxicity. Mechanistic investigations revealed that Sigmar1 modulates mitophagy by influencing the stability of PINK1 at the mitochondria and the recruitment of Parkin to these organelles. Sigmar1 knockdown resulted in reduced PINK1 stability and impaired translocation of Parkin to depolarized mitochondria. These findings indicate that Sigmar1 functions upstream of the PINK1/Parkin pathway, thereby promoting mitophagy through the stabilization of PINK1 and the activation of Parkin. In addition, a distinct mechanism was recently identified by Gao et al. [82] in a LPS-induced acute lung injury model. In this context, Sigmar1 was shown to regulate mitophagy via SIRT3-mediated deacetylation of ATP5F1A, the alpha subunit of mitochondrial ATP synthase F1. SIRT3 is a mitochondrial NAD^+^-dependent deacetylase that is involved in the regulation of mitochondrial metabolism and oxidative stress. LPS exposure was found to increase acetylation at lysine residue 498 of ATP5F1A. Activated Sigmar1 upregulated SIRT3 expression by inhibiting LRRK2, leading to deacetylation of ATP5F1A and the subsequent initiation of PRKN/Parkin-dependent mitophagy. Collectively, these studies demonstrate that Sigmar1 regulates mitophagy through at least two distinct mechanisms: one involving the stabilization of PINK1 and activation of Parkin to support the classical mitophagic pathway; and another involving SIRT3-mediated deacetylation of ATP5F1A, a mechanism that couples mitophagy to mitochondrial energy metabolism.

Lipophagy is the selective degradation of intracellular lipid droplets via the autophagosome–lysosome pathway. In this key process, coat proteins on the lipid droplet surface are recognized by autophagic receptors, such as PNPLA2 or PLIN2/3, thereby mediating the engulfment of these droplets by autophagic membranes and their subsequent delivery to lysosomes, where degradation is ultimately completed by acid lipases [83,84]. As first reported by Oyer et al. [29], lipophagy is specifically triggered by the inhibition of Sigmar1 in prostate cancer cells. Lipid droplets are degraded via lipophagy, and this process is potentially modulated by Sigmar1 through ER-lipid droplet interactions. However, the precise relationship between Sigmar1 and autophagy-related genes, such as ATG7 and MAP1S, remains to be further elucidated. The cellular buffering capacity against ROS is compromised by this aberrant and massive degradation of lipids, precipitating severe metabolic dysregulation and oxidative damage, which ultimately exerts a tumor-suppressive effect.

Aggrephagy is the selective autophagic clearance of misfolded proteins and their aggregates. It is typically mediated by receptors such as p62/SQSTM1, which recognizes ubiquitinated proteins via UBA domains and anchors them to autophagosomes for clearance through interactions with LC3 via LIR motifs [85]. In models of dHMN, mutations in Sigmar1 drive the formation of proteotoxic aggregates, which evade efficient degradation by the autophagic machinery. Although the exact mechanisms remain elusive, this accumulation is postulated to result from overly rapid aggregation kinetics or the overloading of autophagic pathways. Nevertheless, this aberrant aggregation is notably attenuated following treatment with the Sigmar1 agonist SA4503 [86,87]. Collectively, these observations imply that aggrephagy is potentially modulated by Sigmar1 via specific pathways, though the precise mechanisms warrant further exploration. In Aβ42-expressing *Caenorhabditis elegans* models, thioflavin S-positive Aβ42 aggregates are diminished and the paralysis phenotype induced by protein aggregation is significantly delayed following treatment with the Sigmar1 agonist ANAVEX2-73, an effect mediated by the enhancement of autophagy. However, given that these observations are currently confined to nematode models, whether identical therapeutic effects can be replicated in human neuronal models remains a subject for further investigation [88].

ER-phagy serves as a critical mechanism for maintaining ER homeostasis, whereby stress is alleviated through the selective degradation of damaged or redundant ER fragments. This pathway is reliant on specific ER-resident receptors, such as FAM134B, by which membrane curvature changes are sensed and the autophagic machinery is recruited [89,90]. Chronic ER stress has been extensively validated as a crucial trigger for the induction of ER-phagy [91,92]. Currently, direct experimental evidence establishing the involvement of Sigmar1 in selective ER-phagy has yet to be provided. However, Sigmar1 has been characterized by many studies as a pivotal regulator of the ER stress response. For instance, as demonstrated by Shafiul et al. [93], the overexpression of Sigmar1 in NRCs was observed to decrease CHOP levels, elevate phosphorylated IRE1α levels, and enhance the nuclear translocation of XBP1s. These observations indicate that ER stress-induced cardiomyocyte apoptosis is antagonized by Sigmar1 via the activation of the IRE1α-XBP1s signaling pathway, which consequently suppresses CHOP expression.

In summary, Sigmar1 regulates autophagy at multiple levels: it activates AMPK-dependent initiation, promotes LC3 lipidation for autophagosome elongation, facilitates TFEB nuclear translocation and GABARAP-mediated autolysosome fusion, and modulates mitophagy, lipophagy, and aggrephagy. Direct evidence for ER-phagy remains unavailable. However, Sigmar1 has been characterized by many studies as a pivotal regulator of the ER stress response. Taken together, it can be noted that autophagy may be regulated by Sigmar1 through distinct signaling pathways. The use of various cell models across different studies, along with the distinct cellular states and environments, as well as the interaction of Sigmar1 with different proteins at the MAM may all serve as factors responsible for the differential effects of Sigmar1 on autophagy.

## 5. Research Progress of Sigmar1 and Its Ligands in Disease Models

In recent years, significant progress has been made in understanding the role of Sigmar1 in regulating autophagy and other cellular processes and its involvement in the pathogenesis of various diseases.

### 5.1. The Role of Sigmar1 in Cancer

The Sigmar1 has been shown to exert pleiotropic effects in various cancer models through its involvement in autophagy and other cellular processes. Autophagy has been implicated in the survival and progression of advanced-stage tumors. This is supported by evidence that exposure to stress conditions, such as hypoxia or nutrient deprivation, induces autophagy, thereby promoting tumor cell survival [94]. In addition, autophagy contributes to meeting the high metabolic and energetic demands of proliferating tumor cells by recycling intracellular components, thereby generating metabolic precursors that facilitate tumor growth [95]. It has been reported that in uveal melanoma, Longhitano et al. [96] demonstrated that following treatment with the Sigma1 receptor ligand (+)-pentazocine, a significant increase in autophagic cells was detected by flow cytometry, indicating that autophagy was significantly induced. However, the underlying mechanism and whether autophagy serves as a survival response to cytotoxic chemotherapy remain unclear. In prostate cancer, it was revealed by Oyer et al. [29] that after shRNA-mediated knockdown or pharmacological inhibition of Sigmar1 with IPAG, LC3B levels were increased and lipid droplet accumulation was decreased, indicating that this was due to the triggering of lipophagy, which modulates lipid droplet metabolism, thereby compromising the oxidative stress buffering system essential for cancer cell proliferation. However, the interaction mechanism between Sigmar1 and lipophagy remains to be further elucidated. In terms of ligands, an elevation in LC3B levels along with the induction of ER stress and autophagy has been shown to be triggered by the inhibition of Sigmar1 with IPAG, resulting in the selective degradation of immature PD-L1, thereby promoting apoptosis and hindering tumor growth [97]. Consistently, it was also reported by Joel et al. [98] that an increase in LC3B levels is induced by the Sigmar1 inhibitor IPAG, thereby facilitating the selective killing of cancer cells via the ER stress–autophagy axis. However, the ligand selectivity and kinetic mechanisms remain to be further elucidated. Given these roles, Sigmar1 antagonists are considered promising therapeutic candidates, although research in this area remains relatively limited. Conversely, the effects of Sigmar1 agonists remain a subject of debate. While PRE-084 has been reported to promote lung cancer cell growth through cytokine-dependent and receptor-mediated pathways [99], it has been discovered that the migration of cancer cells can be inhibited by the agonist 4-IBP via Sigma1-dependent actin remodeling and the downregulation of Rho GDI. Concurrently, chemosensitivity is enhanced through non-apoptotic and non-ER stress pathways, thereby offering novel therapeutic insights for the treatment of glioblastoma [100]. Beyond autophagy modulation, Sigmar1’s regulation of ion channels significantly impacts cancer metabolism. For example, constitutive calcium influx was demonstrated by Gueguinou et al. [101] to be significantly reduced by Sigmar1 deficiency. Their findings suggest that calcium entry is enhanced through the direct interaction of Sigmar1 with calcium-activated potassium channels, such as SK3, and voltage-independent calcium channels, such as Orai1, ultimately driving the proliferation and invasion of breast and colorectal cancer cells. Collectively, these studies reveal Sigmar1’s pivotal role in cancer therapy and metabolism.

### 5.2. The Role of Sigmar1 in Neurodegenerative Disease

Neurodegenerative diseases such as AD, ALS, and PD have exhibited an increasing global incidence in recent years, significantly impacting patients worldwide. Numerous studies suggest that these diseases are associated with neuronal structural damage, autophagy dysregulation, ion channel dysfunction, and intracellular signaling pathways alterations, with treatment strategies focusing on restoring neuronal structural integrity [102]. The functional relationship of Sigmar1 with neurodegenerative diseases has remained a subject of ongoing investigation. In a model of PD, the Sigmar1 agonist PRE-084 was found to promote neuronal survival by enhancing mitophagy via the PINK1/Parkin pathway [81]. Furthermore, Lin et al. [71] demonstrated that in models of ALS and FTD, the Sigmar1 agonist fluvoxamine restores TFEB-dependent autophagy by stabilizing the nucleoporin POM121 and subsequently promoting TFEB nuclear translocation. Moreover, in ALS models, PI3K/Akt and MAPK/ERK signaling is activated by the Sigmar1 agonist SA4503 through the promotion of Akt and ERK1/2 phosphorylation, thereby effectively attenuating mouse motor neuron death [103]. In AD research, Penke B et al. [104] reported that Sigmar1 levels showed no significant changes during normal aging, but were markedly decreased in brain tissues and post-mortem samples from AD patients, suggesting that Sigmar1 dysfunction plays a crucial role in β-amyloid (Aβ)-induced neuronal loss [105]. Consequently, Sigmar1-activating drugs are considered to possess anti-amnesic and neuroprotective effects against AD and related disorders. In ALS research, motor neurons have been shown to express the highest Sigmar1 levels in the central nervous system, where Sigmar1 may facilitate ion transport through potassium channels [106]. This suggests that slowing ALS progression may be achievable by reducing motor neuron excitability. Collectively, these findings suggest that Sigmar1 agonists hold promise for neuroprotection and disease progression attenuation. Appropriate therapeutic targeting of Sigmar1 may offer significant potential for neurodegenerative disorders.

### 5.3. The Role of Sigmar1 in Cardiovascular Disease

Cardiovascular diseases including hypertension, atherosclerosis, and heart failure have exerted widespread and severe impacts on human health, representing major contributors to increasing global morbidity and mortality. Recent studies indicate that Sigmar1 plays a crucial role in the pathogenesis of cardiac hypertrophy, heart failure, ventricular remodeling, and other cardiac pathologies [107]. Qu et al. [108] first demonstrated in a cardiac fibrosis model that Sigmar1 activation inhibits cardiac fibroblast activation by modulating autophagic flux. Mechanistically, the Sigmar1 agonist fluvoxamine was found to activate Sigmar1, thereby suppressing the ER stress IRE1/XBP1 pathway and restoring impaired autophagic flux, ultimately leading to the inhibition of cardiac fibroblast activation and extracellular matrix deposition. In a model of myocardial reperfusion injury, Zhao et al. [109] reported that Akt confers cardioprotection by activating eNOS to produce NO, which subsequently activates mitochondrial KATP channels in a cGMP-dependent manner. Treatment with the Sigmar1 agonist PRE-084 was shown to significantly increase p-Akt and p-eNOS expression, indicating the cardioprotection of PRE-084 through Akt-eNOS pathway activation [110]. In atrial fibrillation models, autonomic dysfunction was ameliorated, neural remodeling was attenuated, and the expression of Sigmar1 and ion channel proteins was upregulated by chronic administration of the Sigmar1 agonist SA4503, which in turn reduced sympathetic nerve activity and lowered susceptibility to atrial fibrillation [111]. Conversely, sympathetic activity was heightened and cardiac dysfunction was further exacerbated by the Sigmar1 antagonist BD1063 [112]. Collectively, these findings suggest that Sigmar1 agonists possess cardioprotective potential across various cardiovascular disease models, whereas antagonists may aggravate pathological progression, thus providing a clear research trajectory for the development of Sigmar1-targeted cardioprotective therapeutics.

### 5.4. The Delivery System of Sigmar1 Agonists and Antagonists in Different Diseases

Research on the delivery of Sigmar1 agonists and antagonists remains in its early stages. One study demonstrated that encapsulation of ALA derivatives in PLGA nanoparticles confers agonist activity [113]. In addition, surface modification of nanoparticles with ligands such as anisamide has been shown to enhance targeting to tumor cells that overexpress Sigmar1 [114]. Van Waarde et al. further confirmed the potential of Sigmar1 ligand-conjugated nanoparticles for the targeted delivery of anti-tumor agents in animal models of cancer [115]. Future efforts should focus on the development of optimized delivery systems to improve targeting efficiency and facilitate clinical translation.

**Figure 3:**
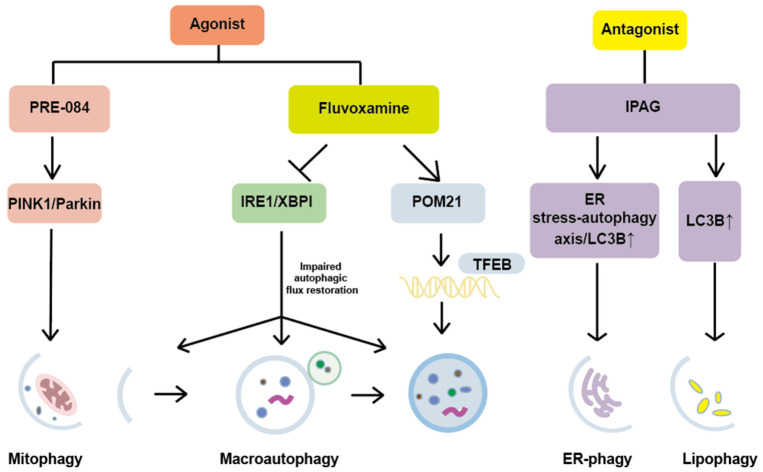
Mechanisms of Autophagy Regulation by Sigmar1 Ligands.

In summary, the roles of Sigmar1 and its ligands have been extensively studied across various disease models (Table 1). It can play a role in diseases by affecting autophagy and other pathways, with its role through autophagy by its ligands is shown in Figure 3. In cancer, pleiotropic effects are exerted by Sigmar1 through the modulation of autophagy, lipid droplet metabolism, calcium signaling, and PD-L1 degradation. Therapeutic potential has been shown for both agonists and antagonists, although their roles remain context-dependent. In neurodegenerative diseases such as AD, ALS, and PD, neuroprotection is consistently promoted by Sigmar1 agonists via the enhancement of mitophagy, the restoration of TFEB-dependent autophagy, and the activation of pro-survival pathways. In cardiovascular diseases including cardiac fibrosis, ischemia/reperfusion injury, and atrial fibrillation, autonomic dysfunction is ameliorated, sympathetic activity is reduced, and cardiac function is improved by Sigmar1 agonists through regulation of autophagic flux and the Akt-eNOS pathway, whereas pathology is aggravated by antagonists. Delivery systems for Sigmar1 ligands, for example PLGA nanoparticles and anisamide-modified carriers, remain in early stages of development but hold promise for enabling targeted therapy. Collectively, Sigmar1 represents a promising therapeutic target. However, its effects are highly dependent on disease context, cell type, and the specific ligand employed. Although no definitive mechanistic explanation has been available in the current literature, the observed differences are likely attributable to variations in cell type, cell state such as stress level and metabolic condition, and the expression profile of Sigmar1 itself across different models. Depending on the cellular context, autophagy may be either promoted or suppressed by the regulation of Sigmar1 and interactions with distinct partner proteins, for example IRE1, TFEB, and PINK1, thereby leading to opposite functional outcomes, such as cell survival versus cell death. Future studies are needed to clarify these context-dependent mechanisms.

## 6. Summary and Outlook

In recent years, significant progress has been made in elucidating the regulatory role of Sigmar1 in autophagy, encompassing molecular mechanisms, disease models, and ligands. At the molecular level, Sigmar1 has been shown to modulate autophagy at multiple stages: during initiation, its effects are mediated through the AMPK/mTOR pathway; in the elongation phase, it binds to LC3B mRNA to promote the translation and lipidation of LC3; and at the degradation stage, it not only facilitates TFEB nuclear translocation by interacting with the nucleoporin POM121 to activate autophagy-related genes, but also directly engages with GABARAP, a protein involved in autophagosome–lysosome fusion. For mitophagy, Sigmar1 regulates this process through two distinct mechanisms. On one hand, it promotes the classical autophagic pathway by maintaining PINK1 stability and facilitating Parkin activation. On the other hand, it modulates mitophagy coupled to mitochondrial energy metabolism via SIRT3-mediated deacetylation of ATP5F1A. For lipophagy, Sigmar1 inhibition triggers excessive lipid droplet degradation. For aggrephagy, Sigmar1 activation attenuates toxic protein aggregates. For ER-phagy, direct evidence is lacking, but Sigmar1 can modulate ER stress which possibly has an impact on ER-phagy. Sigmar1 has been shown to contribute to the pathogenesis of cancer, neurodegenerative disorders and cardiovascular diseases across diverse disease models, partly through its regulation of autophagy and other cellular processes. Pharmacological modulation of Sigmar1 activity has been shown to exert neuroprotective and anti-proliferative effects in preclinical models, thereby offering novel therapeutic avenues for these diseases. These findings are accelerating the translation of Sigmar1 agonists and antagonists into clinical applications, with promising potential in neuroprotection, oncology and cardioprotection.

Despite these advances, several limitations remain and warrant further investigation. First, the molecular mechanisms underlying Sigmar1-mediated autophagy require further elucidation. The interplay and hierarchical relationships among the multiple signaling pathways modulated by Sigmar1 remain poorly understood, hindering the prediction of global effects of Sigmar1-targeted drugs. Furthermore, the specific molecular targets through which Sigmar1 acts at each stage of autophagy and which of these stages are most critical for therapeutic intervention have yet to be fully elucidated, leaving a gap in the rational design of combination therapies. Moreover, the specific mechanisms underlying the distinct phenotypes of Sigmar1 across different diseases and its opposing regulatory effects on autophagy in certain contexts remain to be elucidated. Second, current disease models often fail to fully recapitulate the complexity of human pathology. Many studies rely on gene knockout or acute injury models, which do not adequately reflect the chronic and age-related progression of diseases such as neurodegeneration and cardiovascular diseases. In the context of autophagic regulation, the fundamental distinction between pharmacological activation of the receptor and alterations in its protein expression levels is frequently conflated. As a molecular chaperone, the physiological function of Sigmar1 is dependent on intricate protein homeostasis. Receptor activation elicited by small-molecule ligands fundamentally entails a short-term conformational change, followed by the acute release of its chaperone function upon dissociation from BiP. Conversely, alterations in expression levels induced by genetic overexpression or knockout involve the long-term remodeling of cellular chaperone capacity under chronic disease conditions. In the current literature, the short-term fluctuations in autophagic flux following acute drug administration are often conflated with the long-term compensatory phenotypes observed in gene-knockout models, thereby overlooking the profound spatiotemporal disparities between the two. Third, limitations associated with currently available research tools persist. Commonly used Sigmar1 ligands, such as fluvoxamine, lack sufficient selectivity, complicating the exclusive attribution of observed phenotypes to Sigmar1. For example, fluvoxamine is fundamentally a selective serotonin reuptake inhibitor, whereas ligands such as PRE-084 exhibit pharmacological cross-affinity for other targets, including Sigmar2 and certain ion channels. Due to this deficiency in ligand specificity, it is rendered exceedingly difficult for the observed autophagic phenotypes to be definitively and exclusively attributed to Sigmar1, thereby posing a severe risk of off-target effects. In addition, conventional assays, including immunoblotting for LC3, provide only static measurements and cannot quantitatively monitor autophagic flux in living cells.

To address these challenges, future research should prioritize the following directions. First, the application of highly selective tools and advanced techniques, such as live-cell imaging, will be essential for delineating pathway hierarchies and dynamically localizing Sigmar1’s sites of action under specific physiological and pathological conditions. Second, the use of patient-derived induced pluripotent stem cell models and humanized animal models will enable more precise modulation of Sigmar1 and improve the translational relevance of preclinical findings. Third, the development of next-generation, highly selective Sigmar1 ligands is critical for achieving precise pharmacological control. In addition, innovative technologies capable of real-time, in situ monitoring of autophagic flux are needed to capture the dynamic nature of this process. Collectively, these multifaceted approaches will provide a comprehensive understanding of the intricate regulatory network by which Sigmar1 governs autophagy, ultimately facilitating its translation into effective clinical strategies.

## Figures and Tables

**Table 1:** The Role of Sigmar1 in different diseases.

Disease Category	Disease Name	Sigmar1 Alteration	Mechanism	Outcome	References
**Cancer**	Uveal melanoma	Sigmar1 ligand (+)-pentazocine	Increase the autophagic cells	Induces autophagy and apoptosis, promotes proliferation	[96]
	Prostate cancer	Sigmar1 knockdown or IPAG	Triggers lipophagy, compromising redox buffering	Disrupts redox homeostasis, inhibits proliferation	[29]
	Breast and colorectal cancer	Sigmar1 deficiency	Loss of interaction with SK3 and Orai1 reduces Ca^2+^ entry	Reduces constitutive Ca^2+^ influx, inhibiting proliferation and invasion	[101]
	Lung cancer	PRE-084 (agonist)	Cytokine-dependent, receptor-mediated pathways	Promotes cell growth	[99]
	Glioblastoma	4-IBP (agonist)	Sigmar1-dependent actin remodeling and Rho GDI downregulation; non-apoptotic, non-ER stress pathways	Inhibits migration, enhances chemosensitivity	[100]
**Neurodegenerative disease**	Parkinson’s disease	PRE-084 (agonist)	PINK1/Parkin-mediated mitophagy	Enhances mitophagy, promotes survival	[116]
	ALS and FTD	Fluvoxamine (agonist)	Stabilizes POM121, promotes TFEB nuclear translocation, restores autophagic pathway	Ameliorates autophagic dysfunction	[71]
	ALS models	SA4503 (agonist)	Promotes Akt and ERK1/2 phosphorylation	Reduces mutant SOD1-induced motor neuron death	[103]
	Alzheimer’s disease	Sigmar1 decrease	Sigmar1 dysfunction	Contributes to Aβ-induced neuronal loss	[104,105]
	ALS	High Sigmar1 expression in motor neurons	May facilitate ion flux via K^+^ channels	Reducing excitability may slow progression	[106]
**Cardiovascular disease**	Cardiac fibrosis	Fluvoxamine (agonist)	Activates Sigmar1, suppresses IRE1/XBP1 pathway, restores autophagic flux	Inhibits fibroblast activation and ECM deposition	[108]
	Myocardial reperfusion injury	PRE-084 (agonist)	Activates Akt-eNOS signaling	Cardioprotection	[109,110]
	Atrial fibrillation	SA4503 (agonist)	Upregulates Sigmar1 and ion channels, attenuates neural remodeling	Reduces sympathetic activity and AF susceptibility	[111]

## Data Availability

No new data were created or analyzed in this study.

## Abbreviations

The following abbreviations are used in this manuscript:

Sigmar1	Sigma-1 receptor
MAM	Mitochondria-associated endoplasmic reticulum membranes
ER	endoplasmic reticulum
BiP	Binding immunoglobulin protein
AMPK	AMP-activated protein kinase
LC3	Microtubule-associated protein 1 light chain 3
Vps34	class III phosphatidylinositol 3-kinase
ULK1	Unc-51-like autophagy activating kinase 1
TSC2	Tuberous sclerosis 2
PI3P	phosphatidylinositol 3-phosphate
GABARAP	γ-aminobutyric acid receptor-associated protein
TFEB	transcription factor EB
PNPLA2	Patatin-like phospholipase domain-containing protein 2
PLIN2/3	Perilipin2/3
ROS	Reactive Oxygen Species
MAP1S	Microtubule-associated protein 1S
CHOP	C/EBP Homologous Protein
FAM134B	Family with sequence similarity 134, member B
mTORC	mechanistic target of rapamycin complex
LAMP2A	lysosome-associated membrane protein type 2A
ARMS	Ankyrin Repeat-rich Membrane Spanning protein
ESCRT	Endosomal Sorting Complex Required for Transport
dHMN	distal hereditary motor neuropathy
PI3K	phosphatidylinositol 3-kinase
PI	phosphatidylinositol
PE	phosphatidylethanolamine
SNARE	Soluble N-ethylmaleimide-sensitive factor Attachment protein Receptor
ALS	Amyotrophic Lateral Sclerosis
PD	Parkinson’s disease
FTD	frontotemporal dementia
NO	nitric oxide
ERS	endoplasmic reticulum stress
T1DM	type 1 diabetes
AD	Alzheimer’s disease
ALA	alpha-lipoic acid
LPS	lipopolysaccharide
LRRK2	leucine-rich repeat kinase 2
BDNF	brain-derived neurotrophic factor
MPTP	1-Methyl-4-phenyl-1,2,3,6-tetrahydropyridin
